# Mitigating Composition Variability in Post-Industrial PC/ABS Recycling via Targeted Compatibilization

**DOI:** 10.3390/polym17212848

**Published:** 2025-10-25

**Authors:** Silvia Zanatta, Eleonora Dal Lago, Filippo Dall’Amico, Carlo Boaretti, Alessandra Lorenzetti, Martina Roso, Michele Modesti

**Affiliations:** 1 Department of Industrial Engineering, University of Padova, Via Marzolo 9, 35131 Padova, Italy; silvia.zanatta.5@phd.unipd.it (S.Z.); carlo.boaretti@unipd.it (C.B.); alessandra.lorenzetti@unipd.it (A.L.); martina.roso@unipd.it (M.R.); 2 LaPrima Green Solutions S.r.l., Viale Europa, 46, 36033 Isola Vicentina, Italy; eleonora.dallago@laprimagreensolutions.com; 3 LaPrima Plastics S.r.l., Viale Europa, 46, 36033 Isola Vicentina, Italy; filippo.dallamico@laprimaplastics.com

**Keywords:** PC, ABS, plastic waste, mechanical recycling, compatibilizers, design of experiments

## Abstract

The growing demand for sustainable solutions in the plastics industry has highlighted the need to reintroduce post-industrial polymer waste into high-performance applications. This study focuses on the mechanical recycling of automotive scraps containing variable proportions of polycarbonate (PC), acrylonitrile–butadiene–styrene (ABS), and a commercial PC/ABS blend. After determining the composition of two representative batches, a screening of seven commercial compatibilizers and impact modifiers was performed to improve impact strength. Among them, an ethylene–methyl acrylate–glycidyl methacrylate (E-MA-GMA) terpolymer was identified as the most effective additive. Its influence was further investigated through a mixture design approach, varying the composition of the three polymer phases and the additive content (0–10 wt.%). The resulting response surface model revealed a significant increase in impact resistance in PC-rich formulations with increasing E-MA-GMA content, while ABS and PC/ABS showed more complex trends. Rheological, mechanical, and thermal analyses supported the observed behavior, suggesting improved matrix compatibility and reduced degradation during processing. The proposed model enables the prediction of impact performance across a wide range of compositions, offering a practical tool for the optimization of recycled blends. These findings support the potential of targeted compatibilization strategies for closed-loop recycling in the automotive sector.

## 1. Introduction

The increasing demand for lightweight, high-performance engineering thermoplastics such as acrylonitrile–butadiene–styrene (ABS), polycarbonate (PC), and their blends (PC/ABS) has led to their widespread use in the automotive sector, particularly for structural and aesthetic components that may undergo metallization treatments. Recycling these complex, multi-phase materials—especially after end-of-life—poses a critical challenge for sustainability and resource recovery, as conventional disposal undermines the shift towards a circular economy that emphasizes closed-loop recycling of post-industrial waste [1,2]. Among available approaches, mechanical recycling represents the most straightforward and economically viable route for reintroducing these materials into the production chain [1,2]. However, reprocessing blends composed of incompatible phases often triggers thermal and mechanical degradation, such as polymer chain scission and molecular weight reduction, driven by high processing temperatures, strong shear forces, and mismatched rheology among components [1,3,4]. This typically results in recycled materials with diminished mechanical and rheological performance, especially in Izod impact resistance, which is essential for demanding applications. Greater variability in blend composition exacerbates these effects, severely impacting toughness and usability. Recent findings also indicate that, beyond chain scission, the loss of entanglement density induced by shear deformation during melt processing can play a dominant role in the deterioration of mechanical properties, as reported by Phanthong et al. [5]. This shear-induced disentanglement mechanism explains the decline in toughness even when molecular weight remains nearly unchanged, linking property loss to morphological disruption rather than chemical degradation. In this context, impact modifiers and compatibilizers have been proposed to enhance interfacial adhesion and mitigate performance loss, but their effectiveness depends critically on polymer compatibility, blend composition, and dosage [4,6,7].

Among the most valuable yet problematic fractions of post-industrial automotive waste are chrome-plated PC/ABS components discarded during quality control due to surface or dimensional defects. These heterogeneous mixtures often contain uncontrolled proportions of PC, ABS, and PC/ABS blend, impairing mechanical recyclability. While previous research [1] has demonstrated that mixed PC/ABS streams can be mechanically recycled without pre-sorting, achieving consistent, high-grade mechanical properties, particularly Izod impact strength, remains challenging [1,8]. Consequently, there is a pressing need to develop cost-effective and scalable strategies—such as the use of impact modifiers and compatibilizers—capable of enhancing performance in recycled PC/ABS blends. Reactive additives, especially maleic-anhydride-grafted polymers, have been shown to significantly improve toughness and phase compatibility under controlled conditions [4,9]. However, few studies have considered the practical scenario of waste streams with unknown and fluctuating compositions. This study addresses that gap by evaluating a panel of commercial additives in two industrial scrap mixtures with known compositional variability, combining screening and mixture-design methodologies to assess the impact modifier efficacy in restoring Izod toughness and enabling the realization of closed-loop recycling of automotive PC/ABS wastes.

Numerous studies have investigated the effects of mechanical recycling on PC/ABS systems, focusing on property degradation, thermal stability, and phase compatibility. Reprocessing of PC/ABS blends often leads to significant degradation of PC chains, including shear- and hydrolysis-induced chain scission, while ABS tends to suffer from rubber phase oxidation and interfacial debonding, resulting in reduced toughness and impact resistance [1,10,11]. Specific research has shown that repeated melt reprocessing cycles progressively diminish Izod impact strength, strain at break, and ductility, even if tensile modulus remains largely unchanged [10]. Compatibilization strategies—such as the use of maleic anhydride grafted compatibilizers, epoxy-functional chain extenders, or reactive impact modifiers—have been proposed to enhance interphase adhesion and restore mechanical properties [4,12]. These studies demonstrate that optimized additives can significantly improve impact strength and recovery of toughness in recycled PC/ABS blends [13,14]. However, it has been consistently reported that the efficacy of such additives is highly dependent on the PC/ABS ratio within the blend [10,14]. Few studies address the challenge posed by unsorted post-industrial waste streams with variable and unknown compositions [15]. In an earlier work by the authors [1], an FT-IR and Izod-based method to estimate composition of unknown PC/ABS batches was developed, providing a foundation for more targeted downstream processes. Yet most published studies assume fixed or known blend ratios, leaving a critical knowledge gap regarding the performance of compatibilization strategies under real-world recycling conditions. This study aims to fill that gap by applying a mixture-design approach to systematically investigate additive effects across varying compositions of PC, ABS, PC/ABS, and compatibilizer loading.

This study builds upon a previously developed analytical model to determine the compositions of two real-world batches of PC/ABS industrial waste, referred to as Micronero 1 (MN1) and Micronero 2 (MN2). Seven commercial additives were incorporated at 3 wt.% and 5 wt.% into blends with different proportions of PC, ABS, and PC/ABS to assess their effect on impact resistance, thermal stability, and flow behavior. The initial screening revealed notable differences in performance between the two batches, highlighting the influence of initial composition. Consequently, a second experimental phase was conducted using a statistical Design of Experiments (DoE) approach, focusing on impact resistance as the primary response variable due to its relevance in automotive applications. The most effective additive from the screening was further investigated at concentrations from 0 to 10 wt.% in combination with controlled variations of the three polymer components, enabling the development of a four-component mixture design model. This model allowed the prediction of impact strength across a wide composition range and the identification of optimal additive levels. Overall, the results demonstrate that targeted compatibilization can effectively mitigate variability in post-industrial PC/ABS waste, supporting its reintegration into high-value applications through closed-loop recycling strategies.

## 2. Materials and Methods

### 2.1. Materials

The polymeric materials employed in this work are derived from post-industrial waste collection in the automotive sector, rejected from commercial distribution due to non-compliance with quality specifications. The discarded parts contain a mixture of PC (Makrolon 2405, Covestro AG, Leverkusen, Germany), ABS (Novodur P2MC, INEOS Styrolution Europe GmbH, Frankfurt, Germany), and PC/ABS blend (Bayblend T45, Covestro AG, containing 45 wt.% of PC and 55 wt.% of ABS) mixed together with an unknown composition, variable from batch to batch. These scraps are delivered to a shredding process, and the outcome is a heterogeneous thermoplastic powdery feedstock consisting of PC, ABS, and PC/ABS blend in unknown and batch-dependent ratios. The varying properties of this waste make it impossible to mechanically recycle it into a valuable second raw material with consistent chemical, thermal, and mechanical properties. Therefore, the purpose is to stabilize this variable polymer matrix by using suitable additives.

From this post-industrial polymeric waste, internally identified as “Micronero” (MN), two different batches were selected for this study and named MN1 and MN2. The chemical composition of each batch was determined using a three-equation model that combines FT-IR absorbance peak ratios and notched Izod impact strength, as developed and validated in our previous work based on I-optimal mixture design methodology [1]. The results, reported in Table 1, indicate that MN1 consists of approximately 69 wt.% ABS, 6 wt.% PC and 25 wt.% PC/ABS blend, while MN2 contains 88 wt.% ABS, 3 wt.% PC and 9 wt.% PC/ABS.

To enhance the performance of the recycled blends, a screening study was conducted, using several impact strength modifiers and compatibilizers. These additives were selected based on their chemical structure and established compatibility with the target polymer system, and they were incorporated into the polymer matrix at concentrations of 3 and 5 wt.%. The relatively narrow additive concentration range was chosen based on cost considerations, as recycled materials often require economically viable solutions to compete with virgin alternatives. They are listed as follows:

- Lotader AX8900 (Arkema, Colombes, France) is a random ethylene-methyl acrylate-glycidyl methacrylate (E-MA-GMA) terpolymer employed as an impact modifier in engineering thermoplastics;
- Elvaloy AC1224 (Dow Chemical, Midland, MI, USA) is an ethylene-methyl acrylate copolymer (EMAco) with a 24% weight content of methyl acrylate;
- Paraloid EXL-2650J (Dow Chemical, Midland, MI, USA) is a methyl metacrylate/butadiene-styrene (MBS) core-shell impact modifier employed in polyesters and polyamide blends;
- Europrene SOL THX3300 (Versalis, San Donato Milanese, Italy) is a polymer obtained from the selective hydrogenation of styrene-butadiene-styrene linear block copolymer, with a 30% weight content of styrene and grafted with maleic anhydride (SEBS-g-MAH);
- Dutral CX 2907 (Versalis, San Donato Milanese, Italy) is an ethylene-propylene copolymer grafted with maleic anhydride (EPDM-g-MAH), containing over 50% by weight of ethylene monomer;
- Setabond ABS (Seta Polymers srl, Fontaniva, Italy), a maleic anhydride grafted ABS (ABS-g-MAH) employed for glass-filled ABS of ABS/polyamide blends;
- Setabond SBS (Seta Polymers srl, Fontaniva, Italy), a maleic anhydride grafted SBS (SBS-g-MAH) employed for filled SBS or SBS/polar resin blends.

Following the additive screening phase, one compound emerged as the most effective in enhancing the impact resistance of both recycled batches, MN1 and MN2. To further investigate the influence of polymer matrix composition and additive content on impact performance, three individual polymers—PC, ABS, and a commercial PC/ABS blend—were recovered from post-industrial automotive waste and used as the base components for new blend formulations. The selected compatibilizer, an E-MA-GMA terpolymer, was chosen due to its superior performance in preliminary trials. It was incorporated into the formulations at concentrations ranging from 0 to 10 wt.%. The upper limit was established to balance cost-effectiveness with functional performance, as excessive additive loading is known to induce phase separation and agglomeration, which can negatively affect the dispersion of the compatibilizer and ultimately reduce the blend’s impact resistance.

### 2.2. Methods

#### 2.2.1. Processing

Recycled blend formulations were prepared from MN1 and MN2 powders. These materials were first dried in a dehumidifier (DAC6, Plastic System, Borgoricco, Italy) at 100 °C for 8 h, then dry-blended with selected compatibilizers added at 3 and 5 wt.%. Reactive extrusion was performed using a co-rotating intermeshing twin-screw extruder (Collin ZK25, Maitenbeth, Germany) equipped with a light vacuum to remove volatile compounds during melt processing. The extruder features a screw diameter of 25 mm, a length-to-diameter ratio (L/D) of 36, and a maximum rotational speed of 200 rpm. The two co-rotating screws provides superior blending performance, crucial for achieving polymer compatibilization. The feed rate during extrusion was approximately 1 kg/h. The extrusion temperature profile was set to 200–230–240–250–260 °C, with a constant screw speed of 150 rpm. The extrudate was cooled in a water bath and pelletized. Standard test specimens for mechanical characterization were produced by drying the pellets at 100 °C for 6 h, followed by injection molding using a laboratory-scale injection molding machine (Negri Bossi Canbio V55-200, Cologno Monzese, Italy). The barrel temperature profile was set at 230–240–250–260 °C with a mold temperature of 70 °C. Injection speed ranged from 25 to 45% of the machine’s maximum capacity, and the injection pressure varied between 45 and 70 bar depending on the filling behavior of each formulation. Although optimal processing temperatures differ among ABS, PC, and PC/ABS, a single temperature profile was used to replicate real-world processing conditions, where the exact composition of recycled waste streams is unknown.

Following the identification of E-MA-GMA as the most effective additive for improving impact resistance in both MN1 and MN2 recycled blends, a mixture design study was carried out using a Design of Experiments (DoE) approach. The objective was to evaluate the influence of blend composition and additive content on impact performance, and to identify the optimal formulation. For this purpose, the three base polymer components—ABS, PC, and a commercial PC/ABS blend—were manually separated from post-industrial automotive scraps, shredded, and used to prepare controlled four-component mixtures. The selected compatibilizer, E-MA-GMA, was incorporated into the formulations at concentrations ranging from 0 to 10 wt.%. Before processing, all components were dried in a DAC6 dehumidifier (Plastic System, Borgoricco, Italy) at 100 °C for 8 h. The dried materials were then dry-blended and subjected to reactive extrusion, followed by injection molding under conditions consistent with those previously described.

#### 2.2.2. Characterization

FT-IR spectra were collected using a spectrophotometer (Nicolet™ iS™50, Thermo Fisher Scientific Inc., Waltham, MA, USA), equipped with a Smart ITR accessory and a diamond crystal. The analysis was performed in Attenuated Total Reflectance (ATR) mode and a spectral resolution of 2 cm^−1^.

The melt flow index (MFI) of both the pure and modified materials was measured with a melt flow meter (Instron MF10, Norwood, MA, USA), according to the ISO 1133 standard [16], using a reference set of conditions usually employed for a 45%/55% PC/ABS blend of 260 °C and 5 kg.

Notched Izod impact strength was measured by an impact pendulum (Instron CEAST 9010, Norwood, MA, USA) according to the UNI EN ISO 180 standard [17] to assess the mechanical performance of the material.

Tensile tests for the determination of elastic modulus, tensile strength, and elongation at break were measured with a dynamometer (Galdabini Sun 2500, Cardano al Campo, Italy) equipped with a 25 kN load cell, according to UNI EN ISO 527 standard [18]. The mechanical properties of the injection-molded materials were investigated, testing five samples for each kind of material under controlled conditions (23 °C and 50% RH).

The heat deflection temperature (HDT) was determined according to the adaptation [19] of the standard ASTM D648 [20] to a Q800 Dynamic-Mechanical Analyzer (TA Instruments, New Castle, DE, USA). The samples were tested with a dual cantilever using a heating rate of 2 °C/min from 35 to 120 °C.

The glass transition temperature (T_g_) of the tested materials was measured with the same device and heating rate as for HDT, using a 1 Hz oscillating frequency with a 20 μm fixed strain over the temperature range between 35 and 160 °C.

Finally, morphological characterization of the cryo-fractured and impact-fractured surfaces was carried out using a variable-pressure environmental scanning electron microscope (ESEM, FEI Quanta 200, Thermo Fisher Scientific, Eindhoven, The Netherlands) equipped with a tungsten filament (W). Observations were performed under low-vacuum mode (≈0.6 mbar) at an accelerating voltage of 10–15 kV, with magnifications ranging from 500× to 10,000× depending on the observed feature.

#### 2.2.3. Design of Experiments

The optimization of the selected compatibilizer (E-MA-GMA) content in the recycled polymer mixture was realized with a mixture experiment developed using Design-Expert software (version 22.0, Stat-Ease, Minneapolis, MN, USA), selecting an optimal custom design to accommodate the flexibility required for this study. This design type allows for the inclusion of categorical variables, constrained mixture regions, and customized models tailored to the specific behavior of the system under investigation. In particular, a D-optimal response surface design was employed, as it maximizes the statistical efficiency of the estimated effects and is especially suitable for parameter screening studies. In this context, the D-optimal criterion was used to generate design points concentrated around discrete levels of the additive content. The Coordinate Exchange method was chosen for point selection, as it systematically explores the entire design space to identify the most informative and statistically robust combinations of factors, even when these combinations are not intuitive.

The experimental plan was configured in a single block, as no external sources of variation were present: all materials were sourced from the same production batch, and consistent drying and processing conditions were applied throughout this study. Based on these constraints, the software proposed an initial set of 15 experimental runs, each corresponding to a unique formulation within the mixture space, with additive content ranging from 0 to 10 wt.%. These included 10 runs to model the response surface, 4 runs for lack-of-fit evaluation, and 1 center point. To improve model accuracy and response surface prediction, 17 additional runs were incorporated, as the initial dataset was insufficient to fully capture the behavior of the system. Furthermore, Izod impact data previously collected for pure ABS, PC, and PC/ABS blends—formulated without any additives and reported in our earlier study [1]—were incorporated into the analysis to enrich the response surface description. Lastly, three validation runs were included for each model to assess predictive performance.

## 3. Results

### 3.1. Screening of Compatibilizers

Given the variability in the composition and degradation state of the recycled PC/ABS blends under study and the need to restore adequate impact performance for a closed-loop recycling for the automotive sector, an initial evaluation of additive effectiveness was necessary. Accordingly, the first phase of this study involved the screening of several commercial compatibilizers and impact modifiers, with the goal of identifying the most effective additive for improving Izod impact strength in two distinct recycled matrices composed of PC, ABS, and PC/ABS from automotive waste.

**Figure 1:**
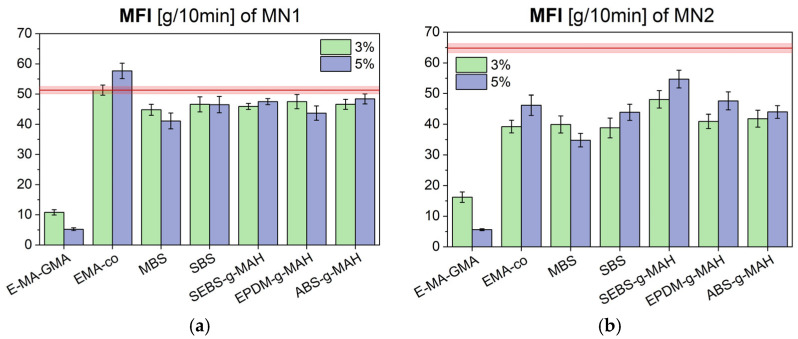
Melt Flow Index (MFI) values for additivated blends based on MN1 (**a**) and MN2 (**b**) compared to their unmodified values (red line).

As an initial step, to assess how the additives affect melt behavior—an important consideration for selecting appropriate processing parameters—the rheological behavior of the modified blends was evaluated by measuring the Melt Flow Index (MFI). The results are presented in Figure 1a,b, where the red line represents the average value for the pristine materials without any additive (MN1 and MN2), and the red semitransparent band represents the interval for the standard deviation; blends containing additives are shown as green and blue bars for 3 wt.% and 5 wt.% of additive, respectively.

Considering the MN1 batch upon the addition of the different additives, in most cases, the value is slightly lower than the pure material and is only marginally affected by the different loading levels. This result is in line with the expectations, upon considering the low concentration of the selected additives, and the possible limited degradation induced by an additional reprocessing step associated with the development of the additivated blends. The EMA-co and E-MA-GMA additives represent the only exceptions. In the first case, a 3% additive is perfectly in line with the starting, pristine material, while with a 5% there is a slight increase above the average value. On the opposite, the addition of E-MA-GMA reveals a dramatic decrease in the value, which is proportional to the content of the additive, with a −80% and −90% decrease, respectively. This result is in line with previous literature [12,21], assuming that the highly reactive glycidyl group of the terpolymer is capable of reacting with the hydroxyl terminal ends of the PC component to act as a chain extender. Given the significant reduction of the MFI, especially with a 5% content, the choice of limiting the concentration of this additive is more than justified. In the case of the MN2 batch, the starting value for the MFI (64.8 ± 1.5 g/10 min) of the pristine material is higher due to the higher ABS content in the blend and possibly a higher extent of material degradation. For this material, the effect of the additives is more marked and, in general, produces a decrease of the MFI down to 40–50 g/10 min. Even for this material, E-MA-GMA shows a marked effect of MFI decrease but less evident at 3 wt.% content in reason of the lower PC content of the blend.

Following rheological evaluation, the blends were processed for mechanical and thermal characterization, including Izod impact, tensile, thermal performance test and Tg evaluation, to assess the effect of each additive on the material properties.

**Figure 2:**
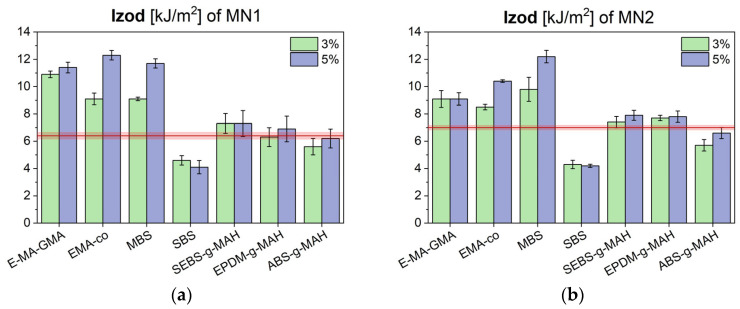
Izod notched impact strength of MN1 (**a**) and MN2 (**b**) blends modified with different compatibilizers and impact modifiers, and compared to their unmodified values (red line).

Izod notched impact strength stands as the key property, since it represents a critical requirement in automotive applications and it is sensitive to both phase compatibility and degradation. The results for the two batches are shown in Figure 2a (MN1) and Figure 2b (MN2).

Among the additives tested, E-MA-GMA, EMAco, and MBS led to a measurable increase in impact resistance, while others—particularly SBS—either had no significant effect or even reduced performance, considering the standard deviation. Additives with similar functional groups exhibited comparable behavior. Specifically, maleic anhydride-grafted modifiers (SEBS-g-MAH, EPDM-g-MAH, and ABS-g-MAH) did not significantly improve impact resistance in either batch. This finding contrasts with the literature, which generally reports good compatibilization of PC/ABS blends using MAH-functionalized copolymers [4,9]. The reduced effectiveness observed here may be attributed to the low PC content in both MN1 and MN2, which limits the extent of interaction between the maleic anhydride and PC chain-end hydroxyl groups. In contrast, the additives producing the most notable increases in impact resistance were those containing methyl acrylate segments. Methyl acrylate copolymers are known to effectively improve ductility in PC and its blends, as reported by Bagotia et al. [22], and are also reported to be effective in ABS-based systems due to their miscibility with SAN copolymers containing acrylonitrile levels typical of ABS. SBS was the least effective additive, likely due to its lack of reactive functional groups and low compatibility with the polar components in the blend. Its toughening effect relies solely on the elastomeric nature of its particles, which absorb and dissipate impact energy through viscous friction. At the low concentrations used in this study, however, SBS particles likely acted as defects rather than reinforcement sites, promoting crack initiation and propagation instead of toughening.

**Figure 3:**
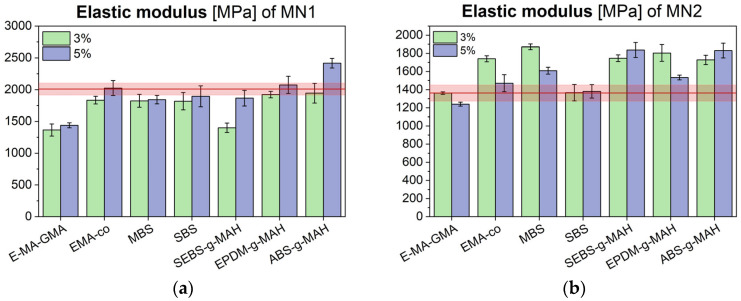
Tensile modulus of MN1 (**a**) and MN2 (**b**) blends modified with different compatibilizers and impact modifiers, compared to their unmodified values (red line).

**Figure 4:**
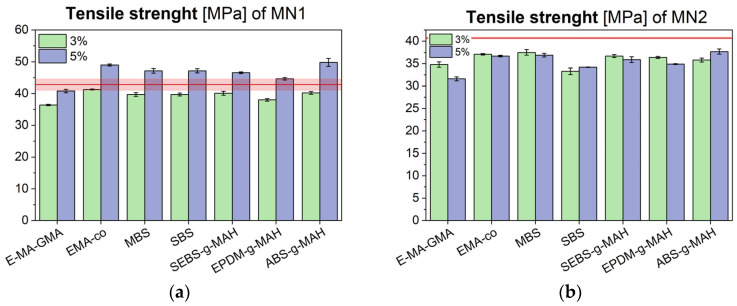
Maximum tensile strength of MN1 (**a**) and MN2 (**b**) blends modified with different compatibilizers and impact modifiers, compared to their unmodified values (red line).

**Figure 5:**
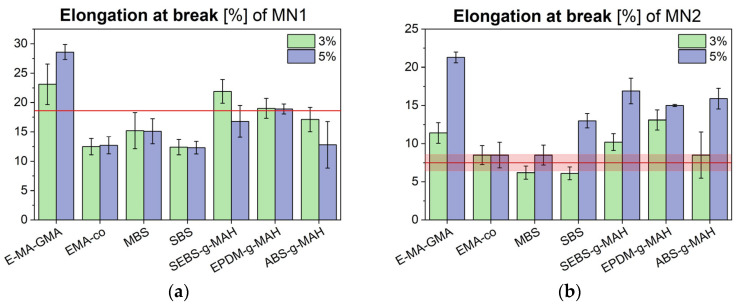
Elongation at break for MN1 (**a**) and MN2 (**b**) blends modified with different compatibilizers and impact modifiers, compared to their unmodified values (red line).

Tensile test results are presented in Figure 3, Figure 4 and Figure 5, showing the elastic modulus, maximum tensile strength, and elongation at break for MN1 and MN2.

The data show that impact modifiers generally increase polymer ductility, which is inversely related to stiffness. As a result, blends with higher elongation at break tended to show lower elastic modulus values. The different rheological behaviour for MN1 and MN2 is reflected in different mechanical performance, especially in terms of tensile modulus. The modulus of MN1 displays a general reduction with most of the additives employed and this is particularly evident, once again, in the case of E-MA-GMA, where the increase in the molecular weight as a consequence of the chemical interaction with the polymer matrix results in a significant drop in stiffness of the material, together with a strong enhancement of elongation at break; these results are consistent with the sharp reduction in MFI observed earlier. In the case of MN2, the tensile modulus of the additivated blends matches or slightly exceeds the reference value, with stiffness values clustering around 1700 MPa. Notably, when comparing the tensile moduli and elongation at break of both MN1 and MN2 after additive incorporation, the additives appear to reduce inter-batch variability, stabilizing the modulus near a common average of approximately 1700 MPa, and elongation at break around 15%. Regarding tensile strength, all modified MN2 blends exhibited a slight reduction, whereas in MN1, only the formulation containing 5% additive showed a modest increase. In cases where a decrease occurred, the reduction was minimal and did not compromise the overall mechanical integrity of the material.

**Figure 6:**
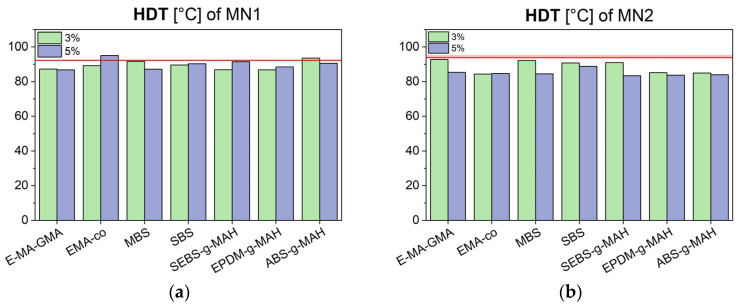
Heat Deflection Temperature (HDT) of MN1 (**a**) and MN2 (**b**) blends modified with different compatibilizers and impact modifiers, and compared to their unmodified values (red line).

Moving to thermal properties, HDT results are shown in Figure 6a for MN1 and Figure 6b for MN2.

All tested additives caused a decrease in HDT compared to the unmodified blends. This reduction is attributed to the inherently low Tg of the modifiers themselves. The decrease became more significant with increasing additive concentration, highlighting the need to monitor this parameter when considering closed-loop recycling in thermally demanding applications, such as automotive parts.

**Figure 7:**
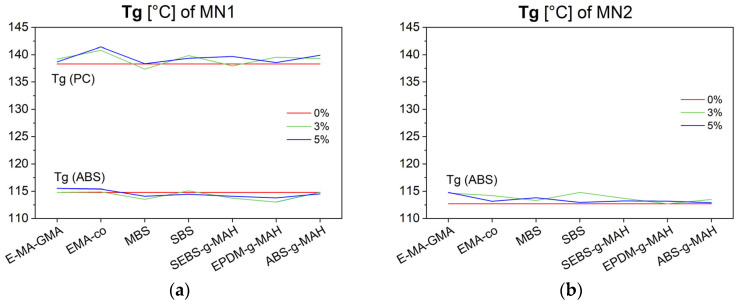
Tg of MN1 (**a**) and MN2 (**b**) blends modified with different compatibilizers and impact modifiers, compared to their unmodified values (red line).

Finally, DMA was performed to assess the Tg and phase compatibility of the blends. The results are presented in Figure 7a for MN1 and Figure 7b for MN2.

MN1 showed two distinct Tg, corresponding to the SAN phase of ABS (between 101 and 116 °C) and PC (between 138 and 163 °C), indicating phase separation and a lack of compatibilization between the components. MN2, on the other hand, exhibited only one transition in the SAN region, consistent with its low PC content (approximately 3 wt.%), as estimated by the previously developed model [1]. The evolution of Tg with additive content in MN1 offers additional insight into compatibilization. An upward shift in the ABS Tg and a downward shift in the PC Tg are indicators of increased miscibility. This shift was observed with 5 wt.% of E-MA-GMA and SEBS-g-MAH (for ABS Tg), and with 3 wt.% of EMAco and MBS (for PC Tg). These trends suggest partial compatibilization effects from these specific additives.

In conclusion, based on the impact resistance data, which represent the primary focus of this study, additives such as SBS, SEBS-g-MAH, EPDM-g-MAH, and ABS-g-MAH were excluded due to their poor or negligible effect. Additives that did not improve blend compatibility, as evidenced by minimal changes in Tg behavior, were also disregarded. From a rheological perspective, E-MA-GMA stood out for its ability to increase the molecular weight of the polymer system, as indicated by a marked reduction in MFI, and therefore it was chosen for a deeper investigation.

A final assessment of the chemical and morphological effect of the E-MA-GMA additive was conducted through Fourier-Transform Infrared Spectroscopy (FT-IR) and Environmental Scanning Electron Microscope (ESEM).

**Figure 8:**
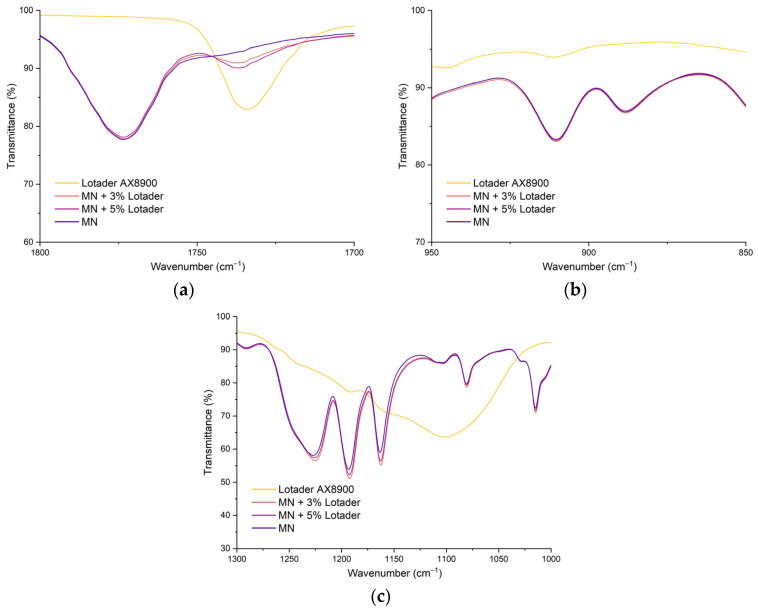
Overlapped FT-IR spectra of MN, MN with 3–5% Lotader and Lotader itself at different wavenumber regions: 1700–1800 cm^−1^ (**a**), 850–950 cm^−1^ (**b**), 1000–1300 cm^−1^ (**c**).

Figure 8 presents the FT-IR spectra focused on three key regions:

- the ~1740 cm^−1^ region, corresponding to the C=O stretching of ester groups,
- the ~910–915 cm^−1^ region, associated with the epoxide ring of the glycidyl methacrylate (GMA) units prior to reaction, and
- the ~1100–1250 cm^−1^ region, which includes C–O–C stretching bands representative of polycarbonate and ABS backbone vibrations.

These spectral regions were selected to monitor both the presence and possible chemical interactions of the E-MA-GMA compatibilizer within the PC/ABS matrix.

The absorption band located near 1740 cm^−1^, characteristic of C=O stretching vibrations of ester groups (from the methyl acrylate units in Lotader AX8900), shows a progressive increase in intensity with the addition of the compatibilizer. While this band is absent in the neat PC/ABS matrix (MN), it becomes clearly visible in the blend containing 3% Lotader and even more pronounced at 5% Lotader, indicating a higher concentration of ester groups. This proportional increase supports the successful incorporation of the E-MA-GMA copolymer into the matrix. Rather than being consumed through complete reaction, a portion of the ester functionalities remains intact after melt blending, suggesting that compatibilization may occur through partial reaction of the GMA epoxy groups, while the acrylate units (containing the ester groups) remain largely unreacted. The presence of this band in increasing intensity thus reflects both the physical presence and chemical contribution of Lotader in the modified blends.

The band centered near 910 cm^−1^ is commonly associated with the epoxide ring deformation of the glycidyl methacrylate (GMA) segment in Lotader [23,24]. In the pure Lotader AX8900 spectrum, a weak but noticeable shoulder is evident around 910 cm^−1^, confirming the presence of intact epoxide functionalities. However, this feature is absent or significantly diminished in the spectra of both MN + 3% Lotader and MN + 5% Lotader, where these spectra are overlapping to the neat matrix curve. The complete disappearance of this epoxide band in the blends strongly indicates that the GMA epoxy rings have undergone chemical reaction during melt processing. The most likely mechanism involves ring-opening reactions with nucleophilic groups, such as –OH from polycarbonate, resulting in covalent grafting and improved interfacial compatibility. This chemical transformation confirms the occurrence of reactive compatibilization rather than mere physical blending.

The spectral region from 1300 to 1000 cm^−1^ includes overlapping absorptions from C–O–C stretching, C–C skeletal vibrations, and aromatic ether linkages, commonly present in both PC and ABS structures. The Lotader AX8900 sample exhibits broader and less intense features in this region compared to the PC/ABS matrix, consistent with its aliphatic and less rigid structure. Upon addition of Lotader (3% and 5%), the spectra of the blends show small but consistent shifts in band positions and relative intensities, particularly around 1150 and 1100 cm^−1^, where ester and ether linkages are typically active. The slight broadening and overlap of bands suggest formation of new bonding environments, likely due to grafting reactions between the PC/ABS matrix and the reactive compatibilizer. These spectral changes support the hypothesis that new covalent C–O–C linkages form between the Lotader and PC/ABS phases, reinforcing the compatibilization effect inferred from mechanical and rheological data.

**Figure 9:**
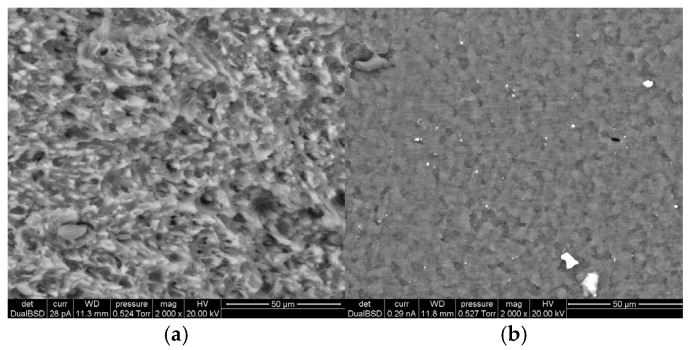

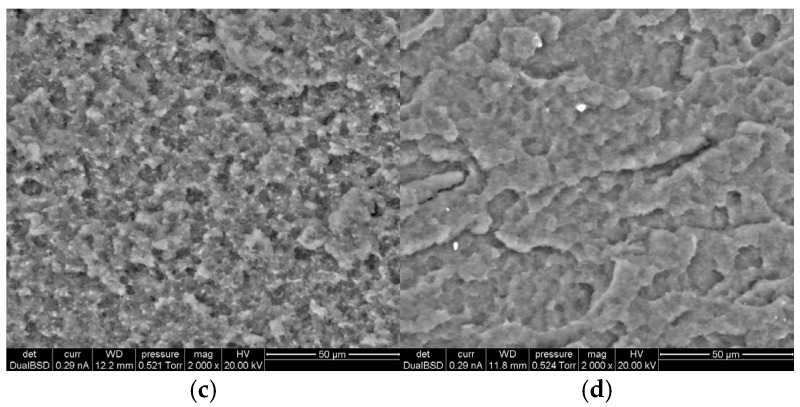
ESEM micrographs of Izod-fracture surface of PC/ABS (**a**) and PC/ABS + 5% Lotader (**c**), and cryogenic-fracture surface of PC/ABS (**b**) and PC/ABS + 5% Lotader (**d**).

In addition to FT-IR evidences, the fracture surface morphology analyzed using ESEM (Figure 9) of the investigated PC/ABS blends provides direct evidence of the influence of the Lotader (E-MA-GMA) compatibilizer on interfacial adhesion and impact behavior under both Izod and cryogenic testing conditions.

The SEM micrographs of the neat PC/ABS blend (Izod—Figure 8a; cryogenic fractures—Figure 8b) exhibit typical brittle fracture features, characterized by visible interfacial voids between the PC matrix and ABS dispersed phase, indicating weak interfacial adhesion and limited energy dissipation capability during impact. At cryogenic temperature, the surface becomes smoother, with numerous microcracks and signs of interfacial decohesion, suggesting that crack propagation occurs predominantly along the PC–ABS interface rather than through the bulk matrix. Similar brittle morphology of PC/ABS systems have been reported by Husaini et al. [25] and Bärwinkel et al. [26], who observed a marked reduction in toughness as the phase interface acts as a preferential crack initiation site under low-temperature conditions.

In contrast, the SEM micrographs of the compatibilized PC/ABS + 5% Lotader blend reveal a rougher and more ductile fracture surface, with evidence plastic deformation zones, especially in the cryogenic-fracture. This morphological evolution is attributed to the reactive compatibilization mechanism of Lotader. The glycidyl methacrylate (GMA) groups in the terpolymer can react with the terminal hydroxyl groups of PC and with residual nitrile or ester groups in the SAN phase of ABS, forming interfacial copolymers that act as molecular bridges between phases [27]. The ethylene–methyl acrylate (E–MA) segments of Lotader, being flexible, also contribute to improved impact resistance by dissipating localized stress through plastic deformation [22].

### 3.2. Formulation Optimization of Recycled Blends with E-MA-GMA

From the screening phase, E-MA-GMA emerged as the most promising additive for enhancing the properties of recycled PC/ABS blends. Although its performance varied between MN1 and MN2, this variability reflects differences in PC, ABS, and PC/ABS content between the two batches. To further explore this composition-dependent behavior, a more detailed study was conducted focusing on the E-MA-GMA additive, with the aim of evaluating its performance across a broader range of blend compositions, using a statistical design of experiments to model and optimize impact resistance, a target property for automotive applications. To support this investigation, a D-optimal mixture design was developed to systematically explore the effect of the additive content across different PC, ABS and PC/ABS compositions. The software initially suggested 15 experimental runs, which were expanded by the authors with an additional 17 formulations to improve the resolution of the response surface. Additional impact data on unmodified blends were incorporated to enhance model reliability across the full 0–10 wt.% additive range. The complete list of experimental runs and the registered Izod impact values is reported in Table 2.

To model the impact behavior of PC/ABS blends modified with E-MA-GMA, a response surface model was developed based on the experimental data listed in Table 2. Since the ratio between the maximum and minimum Izod values was below 10, no transformation of the response variable was required by the software. This was confirmed by the Box-Cox plot, which indicated that the untransformed data were statistically appropriate for modeling. Among the various model types evaluated (linear, quadratic, special cubic, cubic, quartic), the cubic model was selected as the most suitable. It provided the best statistical performance, with a high adjusted R^2^ equal to 0.9693 and a predicted R^2^ of 0.9197, and a non-significant lack-of-fit (*p* = 0.3493), confirming that the model accurately described the experimental data without overfitting. The model also met the adequacy criteria for signal-to-noise ratio (Adeq Precision = 37.1), well above the recommended threshold of 4. Analysis of variance (ANOVA) confirmed the model’s overall significance (*p* < 0.0001), with all model terms—both linear and higher-order interactions—contributing meaningfully to the response. Residuals followed a normal distribution and exhibited random scatter across all diagnostic plots, confirming the validity of the regression assumptions. Cook’s distance analysis revealed no influential outliers, further supporting the model’s robustness.

The final regression equation described the Izod impact strength as a function of the four mixture components: PC (A), ABS (B), PC/ABS (C), and the additive E-MA-GMA (D). The model Equation (1), although complex, captured the nonlinear and interactive effects across the composition space.

Izod = 7.7A + 19.8B + 31.0C − 1099.5D + 33.4AB + 59.9AC + 4945.8AD − 45.3BC + 1246.3BD + 1096.2CD + 169.6ABC − 5383.6ABD − 5015.3ACD + 68.9AB(A − B) + 25.9AC(A − C) − 3469.9AD(A − D) − 73.4BC(B − C)(1)

**Figure 10:**
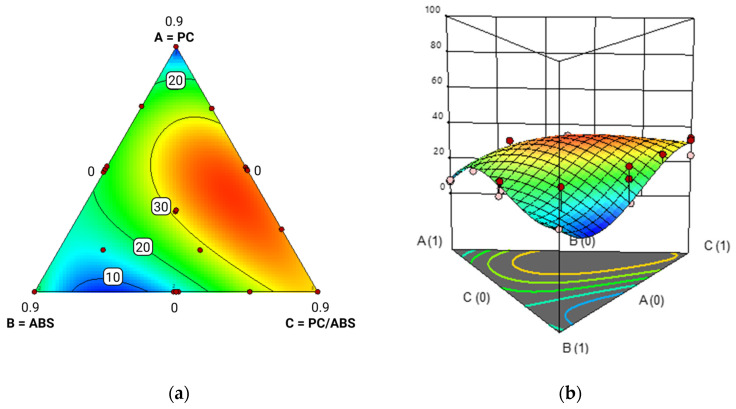
Contour plot (red color denotes the maximum Izod value, while blue represents the minimum value) (**a**) and response surface reporting Izod impact strength on the y-axis (**b**) for PC/ABS blends with 0% additive. ‘A’ stands for ABS, ‘B’ stands for PC and ‘C’ stands for PC/ABS.

**Figure 11:**
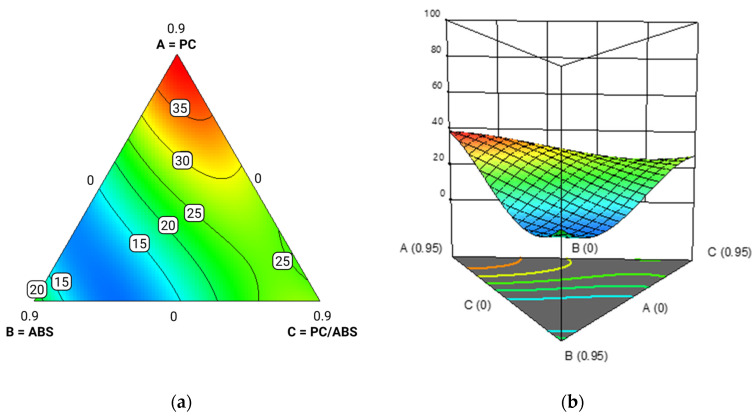
Contour plot (red color denotes the maximum Izod value, while blue represents the minimum value) (**a**) and response surface reporting Izod impact strength on the y-axis (**b**) for PC/ABS blends with 5% additive. ‘A’ stands for ABS, ‘B’ stands for PC and ‘C’ stands for PC/ABS.

**Figure 12:**
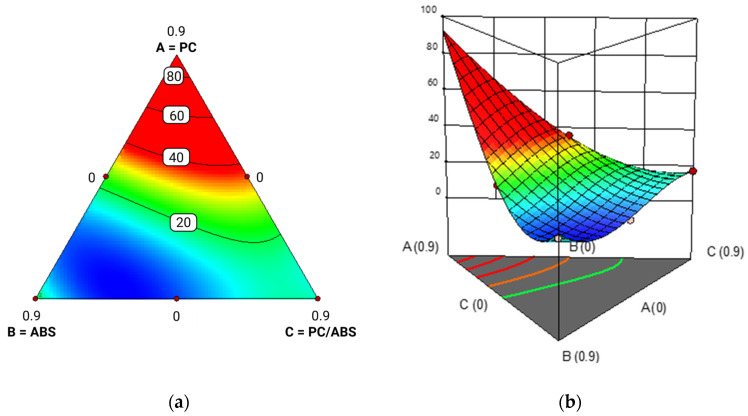
Contour plot (red color denotes the maximum Izod value, while blue represents the minimum value) (**a**) and response surface reporting Izod impact strength on the y-axis (**b**) for PC/ABS blends with 10% additive. ‘A’ stands for ABS, ‘B’ stands for PC and ‘C’ stands for PC/ABS.

To visualize the effects of additive content, contour and surface plots were generated at various additive levels (0%, 5%, and 10%), reported in Figure 10, Figure 11 and Figure 12.

The results showed that polycarbonate (PC) was the most responsive component to the additive, with a significant increase in Izod impact strength. Although the model tended to overestimate values in formulations composed entirely of PC, this region has limited practical relevance due to the typically low PC content in post-industrial waste streams. In contrast, PC/ABS blends exhibited a consistent reduction in impact strength with increasing additive content, indicating limited compatibility or even interference effects. ABS showed an improvement in impact performance up to approximately 7 wt.% additive, beyond which the strength declined, likely due to the formation of segregated additive domains. Overall, higher additive levels accentuated the performance gap among the three components: PC continued to improve, while PC/ABS and ABS showed a plateau or decline beyond certain additive levels. These findings confirm previous observations in the literature that methyl acrylate copolymers like E-MA-GMA are particularly effective at enhancing the toughness of PC-based systems [22]. The additive’s ability to reduce melt fluidity, therefore increasing viscosity, during processing may also help limit thermal and shear degradation, particularly relevant for PC.

Finally, three validation experiments were performed using blend compositions not included in the original model. The experimentally measured Izod values for these runs fell within the 95% confidence intervals predicted by the model, confirming its predictive reliability and suitability for identifying optimal formulations within the design space.

## 4. Conclusions

This study addressed the challenge of optimizing the mechanical performance of post-industrial polymer blends originating from automotive components. These recycled materials, with variable and initially unknown compositions of PC, ABS, and PC/ABS, were first characterized in previous work [1] by the authors. Building on that knowledge, the present research aimed to enhance impact strength using compatibilizers and impact modifiers, thereby enabling the reuse of such waste in closed-loop recycling applications.

An initial screening phase evaluated seven commercial additives at 3 and 5 wt.% concentrations on two industrial batches (MN1 and MN2). Results from Izod impact tests—which stands as the key property for automotive sector—showed a positive trend only with the addition of E-MA-GMA, EMAco, and MBS. Among them, E-MA-GMA stood out for its effectiveness in both batches. Rheological analysis revealed a pronounced decrease in MFI with E-MA-GMA, indicating an increase in melt viscosity and thus molecular weight. Thermal analysis showed a reduction in HDT due to the lower Tg of the additives themselves, while Tg behavior of the blends suggested improved phase compatibility. In contrast, maleic anhydride-grafted modifiers (SEBS-g-MAH, EPDM-g-MAH, ABS-g-MAH) were largely ineffective, likely due to the low PC content in both batches (6 wt.% and 3 wt.%, respectively), limiting MAH reactivity. Similarly, SBS and MBS were less effective, possibly due to their low concentration and tendency to act as defects rather than effective toughening agents.

Given its superior performance, E-MA-GMA was selected for a detailed mixture design study to evaluate its influence as a function of blend composition and additive content (0–10 wt.%). The analysis, performed through a D-optimal design of experiments, produced response surfaces and contour plots that revealed clear trends. Notably, Izod impact strength increased significantly in PC-rich formulations with additive contents approaching 10 wt.%, with predicted values approaching those of virgin PC. This improvement is likely linked to enhanced melt flow behavior, which reduces shear-induced degradation during processing. The additive appears to act as a process aid, preserving the integrity of PC under thermal and mechanical stress.

The generated response surfaces serve as a practical tool for industrial applications, allowing users to tailor the additive content to achieve a desired level of impact performance based on batch composition. Furthermore, the model can predict behavior in untested composition ranges, supporting the future integration of virgin materials for property fine-tuning.

In conclusion, E-MA-GMA proved to be particularly effective in ABS-dominant waste blends, a common finding in automotive post-industrial recycling. Its compatibility with the SAN phase of ABS and favorable processing behavior make it a strong candidate for closed-loop recycling schemes. Future developments should investigate additives optimized for PC-rich blends, which were not represented in the batches tested here. This work also highlights the importance of matching additive chemistry to blend composition, as demonstrated by the limited effectiveness of MAH-based additives in low-PC matrices—despite their proven efficacy in more balanced PC/ABS systems.

## Figures and Tables

**Table 1:** Impact strength and component fractions of the two material batches of scraps.

Batch	Izod [kJ/m^2^]	$w_{\mathrm{ABS}}$	$w_{\mathrm{PC}}$	$w_{\mathrm{PC}/\mathrm{ABS}}$
MN1	6.4 ± 0.25	0.69	0.06	0.25
MN2	7.0 ± 0.17	0.88	0.03	0.09

**Table 2:** DoE run for Izod response as a function of mixture composition.

Run ^1^	$w_{PC}$	$w_{ABS}$	$w_{\mathrm{PC}/\mathrm{ABS}}$	$w_{Additive}$	Izod [kJ/m^2^]
1	0	1	0	0	19.224
2	0	0	1	0	31.266
3	0	0.492	0.508	0	13.550
4	1	0	0	0	7.000
5	0.508	0	0.492	0	33.938
7	0	1	0	0	19.769
8	1	0	0	0	6.802
9	0	0	1	0	30.427
10	0.488	0.512	0	0	21.425
11	0.608	0.157	0.157	0.078	38.313
12	0	0.900	0	0.100	23.427
15	0.405	0.157	0.405	0.033	29.144
16	0.157	0.608	0.157	0.078	9.568
17	0	0.495	0.495	0.010	12.938
18	0	0	0.990	0.010	29.964
19	0.495	0	0.495	0.010	34.899
21	0	0	0.945	0.055	27.219
23	0	0.990	0	0.010	21.657
24	0.315	0.315	0.315	0.055	22.016
25	0.157	0.157	0.608	0.078	21.417
26	0.315	0.315	0.315	0.055	21.709
27	0	0.450	0.450	0.100	7.713
28	0.158	0.382	0.382	0.078	13.328
29	0	0.900	0	0.100	17.318
31	0	0.990	0	0.010	19.943
32	0.450	0	0.450	0.100	35.797
33	0.450	0.450	0	0.100	24.868
35	0	0	0.900	0.100	16.865
36	0.990	0	0	0.010	12.979
38	0	0.943	0	0.057	23.344
39	0	0.990	0	0.010	20.443
41	1	0	0	0	7.511
42	0.488	0.512	0	0	20.219
43	0.500	0	0.500	0	33.464
44	0.495	0	0.505	0	32.194
45	0.500	0.500	0	0	23.537
48	0.757	0.243	0	0	24.745
49	0.328	0.338	0.334	0	30.125
50	0.512	0.488	0	0	21.219
51	0	0.508	0.492	0	14.729
52	0.171	0.672	0.157	0	16.329
53	0	0.240	0.760	0	27.219
54	0.255	0	0.745	0	33.922
55	0	0.508	0.492	0	15.078
56	0.328	0.338	0.334	0	30.297
57	0.748	0	0.252	0	28.339
58	0.333	0.333	0.334	0	31.771
59	0	0.500	0.500	0	14.469
60	0	0	1	0	30.526
61	0.170	0.330	0.500	0	30.656

^1^ Missing numbers are run that were considered outliers.

## Data Availability

The data presented in this study are available on request from the corresponding author. The data are not publicly available due to privacy of the funding company.

## Abbreviations

The following abbreviations are used in this manuscript:

ABS	Acrylonitrile–Butadiene–Styrene
ABS-g-MAH	Maleic anhydride grafted Acrylonitrile–Butadiene–Styrene
DMA	Dynamic Mechanical Analysis
DoE	Design of Experiments
EMAco	Ethylene-methyl acrylate copolymer
EPDM-g-MAH	Ethylene-propylene copolymer grafted with maleic anhydride
E-MA-GMA	Ethylene-methyl acrylate-glycidyl methacrylate
FT-IR	Fourier-Transformed Infrared Spectroscopy
HDT	Heat Deflection Temperature
MBS	Methyl methacrylate/butadiene-styrene
MFI	Melt Flow Index
MN	Micronero
PC	Polycarbonate
SBS	Styrene–Butylene–Styrene
SEBS-g-MAH	Maleic anhydride grafted Styrene–Ethylene–Butylene–Styrene
SBS-g-MAH	Maleic anhydride grafted Styrene–Butylene–Styrene
